# Rabies Vaccine for Prophylaxis and Treatment of Rabies: A Narrative Review

**DOI:** 10.7759/cureus.62429

**Published:** 2024-06-15

**Authors:** Alan D Kaye, Dominique M Perilloux, Elizabeth Field, Cody A Orvin, Spencer C Zaheri, William C Upshaw, Raju Behara, Tomasina Q Parker-Actlis, Adam M Kaye, Shahab Ahmadzadeh, Sahar Shekoohi, Giustino Varrassi

**Affiliations:** 1 Department of Anesthesiology, Louisiana State University Health Sciences Center, Shreveport, USA; 2 School of Medicine, Louisiana State University Health Sciences Center, Shreveport, USA; 3 Department of Pharmacy Practice, Thomas J. Long School of Pharmacy and Health Sciences University of the Pacific, Stockton, USA; 4 Pain Medicine, Paolo Procacci Foundation, Rome, ITA

**Keywords:** next-generation vaccines, post-exposure prophylaxis, one health, vaccination, rabies

## Abstract

Rabies, a millennia-old viral infection transmitted through animal bites, poses a lethal threat to humans, with a historic fatality rate of 100% if left untreated. Louis Pasteur's introduction of the rabies vaccine in 1885 marked a turning point in the battle against rabies, preventing numerous cases. The purpose of this paper is to review the historical development, current challenges, and future prospects of rabies vaccination and treatment, with emphasis on the importance of continued research and collaborative efforts in the quest to eradicate this deadly infection. Historical vaccine development progressed from inactivated to live-attenuated forms, with modern recombinant techniques showing promise. The preventive measures at present primarily involve vaccination, but challenges persist, such as differing safety profiles and immunogenicity among vaccine types. Pre-exposure prophylaxis with a three-dose vaccine series is crucial, especially in high-risk scenarios. Post-exposure prophylaxis combines human rabies immunoglobulin and inactivated rabies virus vaccine. The quest for the next generation of vaccines explores genetically modified and viral vector-based approaches; emerging treatments include gene therapy, virus-like particles, and monoclonal antibodies, offering hope for improved outcomes. Economic barriers to post-exposure prophylaxis, limited education, and awareness challenge rabies control. Cost-effective solutions and comprehensive awareness campaigns are vital for the successful eradication of rabies. More research and collaborative endeavors remain pivotal in the ongoing journey to eradicate rabies, one of the deadliest infectious diseases known to humans, if not met with prophylactic measures.

## Introduction and background

Rabies is a lethal viral infection often transmitted through animal bites or saliva entering through mucosal membranes or broken skin and has been known for thousands of years. It is caused by the bullet-shaped, single-stranded, negative-sense RNA Rhabdoviridae family of viruses, which are carried by both wild and domesticated animals. A viral envelope and ribonucleocapsid core contribute to viral virulence and cause nervous system infection, leading to severe symptoms like hydrophobia and aerophobia. Hydrophobia is defined as fear of water, but for patients experiencing this symptom of rabies, it describes the fear of pain upon swallowing fluids. The pain is caused by spasms of the pharynx due to furious rabies infection, which is characteristic of 80% of human rabies infections. The remaining 20% of cases are paralytic. The presenting symptoms include fever, headache, and fatigue that progress to encephalomyelitis characterized by delirium. The five stages of the disease course are incubation, which can last from days to years; prodrome; acute neurologic illness; coma; and death, thought to be due to the massive inflammatory response in the central nervous system (CNS). Historically, rabies infection was 100% fatal, leading to increased suicide rates in individuals who believed they had contracted the disease. Thanks to Louis Pasteur developing the rabies vaccine in 1885, countless cases have been prevented, especially in developed countries [1].

Vaccination remains the foundation for preventing the infection that causes this viral zoonotic disease in exposed individuals. Along with vaccination, post-exposure prophylaxis includes wound cleaning and rabies immunoglobulin administration. Several types of rabies vaccines are available and utilized today. The issue with the numerous rabies vaccines that have been created is the vaccine candidate's differing safety profiles and immunogenicity. Thus, continued efforts towards rabies vaccine research have focused on improving upon these factors. The limitations of the live-attenuated vaccine include inducing rabies in animals due to mutations in the host and resistant viral capabilities to cause infection [2]. The drawbacks of inactivated vaccines include lower immunogenicity, higher cost, and the requirement of multiple vaccinations during pre- and post-exposure to the rabies virus, which led to the development of adjuvanted vaccines [3]. Research efforts are now geared toward next-generation vaccines, like genetically modified and viral vector-based vaccines, which have limitations, including safety and proper distribution [2].

Preventative measures outside human vaccination include animal vaccination and spreading public awareness [1]. In 2018, the Global Strategic Plan was put in place by international organizations, including the World Health Organization, to eradicate human fatalities due to rabid canines by 2030, highlighting the importance of animal vaccination [2]. Targeted communities for rabies prevention education include high-risk populations like farmers and those who live in areas where rabies is endemic. Additionally, high-risk occupations, such as veterinarians, zookeepers, and forest workers, can receive pre-exposure prophylaxis [4]. This review will discuss the historical development, significance, and challenges surrounding rabies vaccines for prevention and treatment.

## Review

Historical development of rabies vaccines

Beginning as early as the first century BC, various techniques to treat rabies were practiced [2]. The methods, including cauterization, excision, and amputation, among others, were deemed inconsistent and eventually considered unsuitable as treatment methods. But this understanding persisted until Louis Pasteur developed the first rabies vaccine in the nineteenth century [2].

The first generation of vaccines began in 1885 with Louis Pasteur, who used an infected rabbit spinal cord that was inactivated via sun drying to develop a vaccine [2]. The vaccine was able to provide success; however, there were many concerns. The consistency of inactivation of the virus in the vaccine was questioned, given that post-vaccine rabies cases were being reported. Given the development method, large-scale production of Pasteur’s vaccine was also challenging. Using Pasteur’s work, other researchers added their advancements, including adding phenol to inactivate the virus, using baby animal brains to create vaccines as they contained less myelin, using embryonated chicken and duck eggs to make a live attenuated vaccine, and using the cell culture system to enhance the production of a vaccine [2,4,5]. In the modern world, the vaccines used are the modified live vaccine, the inactivated rabies vaccine, and the adjuvanted rabies vaccine [2]. With the advancement of recombinant DNA technologies, new vaccination candidates may soon be available.

Types of rabies vaccines

The first vaccines created to immunize people against the rabies virus contained an inactivated form [6]. These vaccines were initially developed through the isolation of the virus from animals infected with rabies, followed by the inactivation of the virus through the addition of various chemicals [7]. However, this technique led to numerous issues, such as poor antigenicity of the virus and adverse reactions to different components in the vaccine [8]. To overcome these deficiencies, new methods were developed to generate superior vaccines. Improvements in cell culture techniques allowed for the growth of viruses in various cell lines in vitro [9]. After the viruses were cultured, they were extracted from the cells and inactivated via treatment with compounds such as beta-propiolactone and formalin [10]. The inactivated viruses were then administered to people or animals. This stimulates the innate and adaptive immune response, ultimately creating memory B and T cells, providing long-lasting immunological protection against the virus [11]. Vaccines containing the inactivated virus are typically administered via intramuscular injection. The vaccine may be given either pre-exposure or post-exposure, with multiple doses required to achieve maximum immunological protection [12]. Vaccines generated via virus culturing in vitro are safe and efficacious and allow for a high degree of control in growing the virus [9]. Unfortunately, there is a risk that some viruses may not be fully inactivated during development. Additionally, multiple doses of the vaccine are required, which may lead to issues regarding patient compliance [13].

Following the development of vaccines containing inactivated forms of the virus, vaccines for rabies were created containing a live-attenuated form of the rabies virus. These were initially created by passing the rabies virus through numerous cell cultures and selecting nonpathogenic forms of the virus [2]. When vaccines containing this product were administered via intramuscular injection to various animals, a robust immune response was generated, and high levels of antibody titers were recovered in the serum against the rabies virus. However, numerous adverse effects, such as severe tremors and paralysis, were reported [14]. The large number of adverse effects combined with a high sensitivity to fluctuations in temperature resulted in the modified live vaccine no longer being recommended for parenteral administration by the World Health Organization [2,14]. More recently, vaccines containing mutated forms of the rabies virus have been created, greatly reducing virulence [2]. The glycoprotein (g) is crucial for the rabies virus to be pathogenic [15]. Thus, mutations can be induced at sites in the gene that encodes for this protein, rendering the virus no longer pathogenic though still infectious, allowing for a strong immune response to be still generated against it [16]. Vaccines containing mutated forms of the rabies virus may be administered orally and are often given to animals that serve as a reservoir for the rabies virus, such as dogs [2]. The advantages of this vaccination method include the production of a vigorous immune response and few doses required to achieve a high degree of immunological protection. One study showed numerous antibodies against the rabies virus in dogs three years after receiving a single dose of the vaccine [17]. Limitations of the live-attenuated vaccine form are a higher cost than other vaccination methods and a risk of reversion back to the pathogenic form of the virus through back mutations [18,19].

A third method of generating vaccines against the rabies virus involves using recombinant techniques to express proteins of the rabies virus in various vectors such as the vaccinia virus, canary pox virus, and the Orf virus [20-22]. This is done by creating attenuated viral vectors that express the rabies virus glycoprotein. This protein is generally chosen to be expressed in the vector because it is expressed on the surface of the rabies virus. This allows for the generation of antibodies that can bind to glycoprotein, thus preventing rabies virions from entering host cells, as this glycoprotein is critical in mediating cell entry [23,24]. Vaccines containing these recombinant virions are typically only used in animals, with most instances of human administration of this vaccine only being done in the context of clinical trials [21]. However, studies involving animals have shown that this vaccine produces a very strong B and T cell response that offers protection for an extended period, even after just a single administration of the vaccine [25]. Additionally, immunological solid reactions have been documented after both intramuscular and oral administration of this vaccine in animals [25]. The drawbacks of this type of vaccine include its a high cost of production and a limited number of studies of its use in humans (Table 1) [13].

**Table 1:** Characteristics, route of administration, target population, and limitations of different types of rabies vaccines

Vaccine Type	Characteristics	Route of Administration	Target Population	Limitations
Inactivated rabies vaccine [10-13]	Contains rabies virus inactivated via various chemicals	Intramuscular injection	Humans and animals	Many doses required, creating issues with compliance and reduced immune response compared to other methods
Live attenuated rabies vaccine [2, 14, 18, 19]	Contains less virulent form of rabies virus but still capable of replication in host cells	Intramuscular injection or oral administration	Primarily animals, though still given to humans in some parts of the world	Risk of reversion to pathogenic strain and expensive, especially when administered orally
Recombinant-vectored vaccine [13, 20-25]	Contains attenuated vectors expressing the rabies virus glycoprotein	Intramuscular injection or oral administration	Animals with human use restricted to clinical trials	Expensive to produce; limited studies involving its use in humans

Prophylactic use of rabies vaccines

Rabies is a devastating disease that is fatal in nearly 100% of the cases. The primary source of human contraction is through a bite from a rabid animal; in these instances, there are vaccinations available for humans to be taken pre- and/or post-exposure to lower the fatality rate. The effectiveness of the pre- and post-prophylaxis vaccines when taken together is remarkably high, with no reported deaths due to rabies in any individual who has taken both; however, there was one reported death in an individual who had only the pre-exposure immunization with no post-exposure booster [26].

The pre-exposure prophylaxis (PrEP) vaccination is a safe vaccine that can be co-administered with other vaccines in children or can be given separately. The administration of the PrEP vaccination series involves three intramuscular or intradermal injections to prime the patient’s immune system; these injections are done on a standard schedule, and an injection is administered on days 0, 7, and 28 [27]. The purpose of this vaccine is to allow the immune system to exhibit recall when exposed to the virus in the event of a rabid animal bite [27]. Additionally, PrEP administration would decrease the amount of post-exposure vaccination an individual would have to receive in the event of an exposure [27]. This may not limit the cost of overall care per patient, but it most certainly would benefit patients in varying circumstances, such as in remote communities where post-exposure prophylaxis (PEP) is not readily available, in situations where risk of exposure is high and may not be recognized, in places where controlling rabies in the animal reservoir is difficult and where human exposure risk is high, and in occupations where animal interactions are frequent (i.e. bat handlers or zoologists) [27]. PrEP should also be included in the immunization of children in high-risk areas, followed by a booster after one year. 

The administration of the PEP treatment is determined via an assessment based on the risk of infection, the severity of infection, the site of the wound, the appearance of the wound, behavior, the fate of the biting animal, and the vaccination status of the biting animal [26]. An unusual contact with a bat, regardless of location, is a sufficient ground for considering rabies exposure [26]. Rabies exposure is considered as contact of the saliva of a rabid animal to broken skin or intact mucous membrane of the patient [26]. Under these circumstances, PEP is indicated in the patient, regardless of how long before the contact with animal saliva occurred, and the patient will undergo wound care, active immunization, and passive immunization. If a patient was previously immunized, a shorter booster regimen is given along with wound care. There are a multitude of vaccine regimens, both intramuscular and intradermal, that involve variable sites of injection and number of injections, requiring multiple clinical follow-ups to maintain a proper injection schedule [26]. 

Therapeutic applications of rabies vaccines and novel treatment modalities

The current standard for PEP involves a combination of human rabies immunoglobulin (HRIG) and rabies vaccine administration [2]. In a prospective study from January 2021 to December 2021 at the Kempegowda Institute of Medical Sciences (KIMS) Hospital and Research Center, 123 category III animal bite victims provided clinical evidence of the safety and efficacy of HRIG in combination with an entire course of anti-rabies vaccinations for PEP [28]. HRIG provides immediate passive immunity by neutralizing the virus at the site of infection, while the rabies vaccine stimulates the immune system to produce long-lasting active immunity [28]. 

The most used vaccine in combination with HRIG is the inactivated or killed rabies virus vaccine [12]. Immunization against these inactivated vaccines develops over time due to the activation of helper and cytotoxic T cells, allowing for virus-neutralizing antibodies to be produced for long-lasting active immunity. These vaccines are administered globally [29].

The effectiveness of RIG and vaccine therapy in treating rabies infection is well-established, especially when administered promptly after exposure. In an open-label, single-arm study of 12 healthy adult subjects, Hanna et al. found that caprylate/chromatography purified HRIG (RIG-C) produced a rapid increase in rabies-neutralizing antibodies within 24 hours, peaked on day four and maintained through day 21 [30]. However, limitations exist with RIG administration, such as the worldwide availability of RIG, potential allergic reactions, and the need for timely administration immediately following exposure. In the same open-label, single-arm study of 12 healthy adult subjects, a total of 15 adverse events (AE) were noted, including gastrointestinal disorders, injection site pain/nodules, dizziness, and extremity pain, all of which were mild except for one subject who experienced moderate oropharyngeal pain [30]. The efficacy of rabies therapy decreases once clinical symptoms manifest, underscoring the importance of early intervention. Recent research has focused on developing novel treatment strategies composed of molecules that target viral replication at different stages of the rabies virus life cycle and molecules that inhibit some pathways of the innate host immune response [31]. In one study on mice, a cocktail regimen of six compounds was selected for use based on the results of previous studies: caspase-1 inhibitor, tumor necrosis factor (TNF)-α inhibitor, mitogen-activated protein (MAP)-kinase inhibitor, mouse interferon (IFN)-α/β, favipiravir, ribavirin and HRIG [31]. Mannitol was utilized as a blood-brain barrier opener [31]. The results of this study report a statistically significant extension of survival of mice treated with the drug cocktail compared to the survival of mice in the virus control group. They found a significant downregulation of pro-inflammatory molecules (caspase-1 and TNF-α) in the CNS in rhinovirus (RV)-infected mice treated with a combination of drugs, including IFN-α/β [31]. While the traditional combination therapy of RIG and vaccines remains the standard for PEP, ongoing research is uncovering new therapeutic avenues for treating rabies infections.

One approach involves enhancing the immune response using adjuvants or novel vaccine formulations. Adjuvants can boost the immune response, potentially improving vaccine efficacy. A separate animal study on mice revealed that the absence of Toll-like receptor 7 (TLR7), an innate receptor sensing single-stranded viral RNA, led to lower antibody production in mice immunized with rabies virus (RABV) [32]. The results showed that TLR7 deficiency affected the recruitment of germinal center (GC) B cells and led to lessened GC formation, resulting in impaired RABV-neutralizing antibodies (VNA) and an inadequate Th1-based immune response [32]. Novel rabies infection treatments targeting the TLR7 signaling pathway for activation are promising strategies for improving the current efficacy of future rabies vaccines.

Novel vaccine formulations, such as virus-like particles, are being explored for their ability to induce a more potent and more targeted immune response. Another animal study with rabies virus-like particles (RV-VLPs) in mice was conducted using stable cell lines for producing RV-VLPs via lentiviral-based transduction of human embryonic kidney (HEK)-293 cells [33]. This study developed a G protein-expressing cell line (HEK-G) to produce G-containing virus-like particles, evaluated the immunogenicity of these particles in mice, and found that the RV-VLPs were able to induce specific immune responses against rabies virus G protein [33]. These results encourage the study of new VLPs because these particles produced a specific antibody response.

Other advancements in molecular biology and virology have paved the way for potential breakthroughs in more advanced rabies infections. Gene therapy, for example, holds promise for delivering antiviral genes directly to infected neurons by using rAAV-N796, a small interfering RNA (siRNA) that targets the nucleoprotein gene of rabies, preventing the virus from evading the host's innate immune response [34]. Other forms of gene therapy being studied include clustered regularly interspaced short palindromic repeats (CRISPR) or CRISPR-associated protein (Cas9)-based technologies and induced pluripotent stem cells (iPSCs). The iPSC technique is proposed to target the editing of the rabies virus genome, potentially rendering it non-pathogenic, which is more helpful for disease modeling [35]. A combination therapy of the CRISPR/Cas9 system and iPSC method was found to correct erroneous strings in vitro. Gene delivery tools such as adeno-associated virus (AAV), Sendai virus, and episomes can then deliver corrected genes to target organs [35].

**Figure 1:**
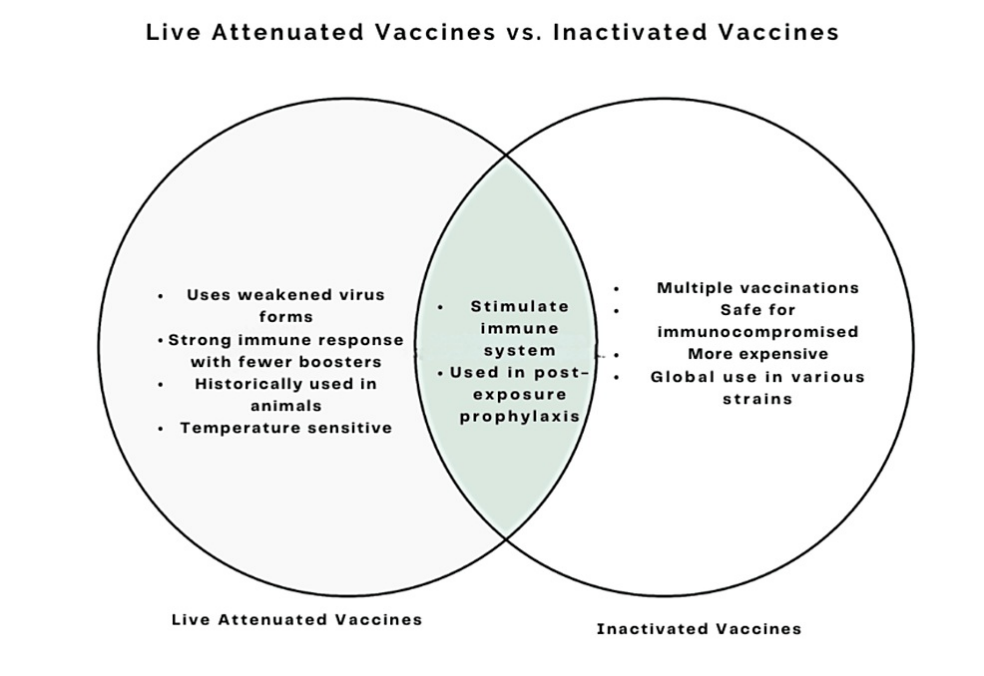
Comparison of live attenuated and inactivated vaccines in rabies prevention. Live vaccines use weakened virus forms and are temperature sensitive, while inactivated vaccines require multiple doses and are globally used. Both stimulate the immune system against rabies and are used in post-exposure prophylaxis.

**Figure 2:**
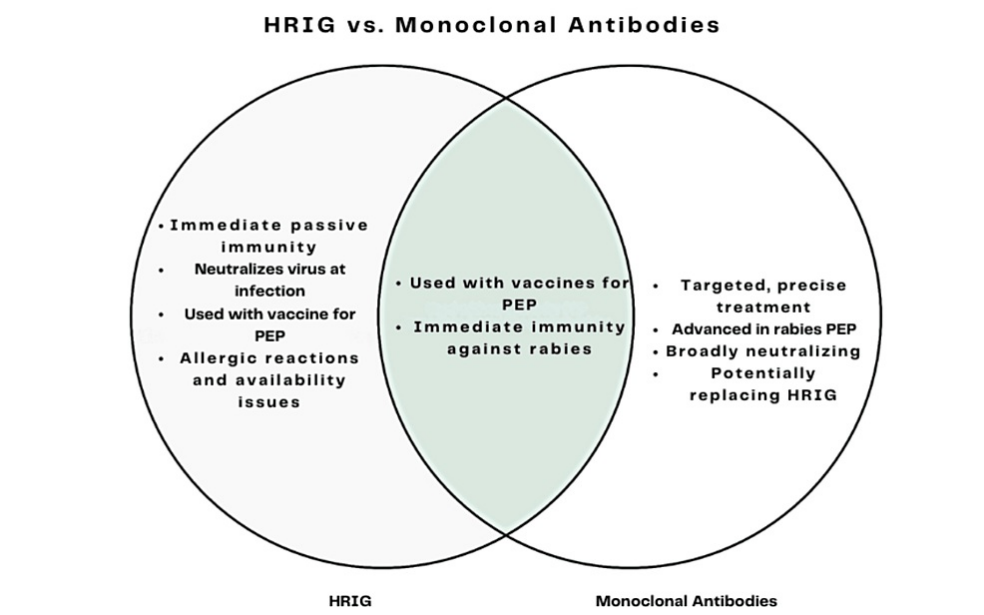
Illustration of the differences and similarities between HRIG and monoclonal antibodies in rabies PEP. HRIG offers immediate immunity but has availability issues; monoclonal antibodies are advanced, broadly neutralizing, and may replace HRIG. Both are used with vaccines for immediate rabies immunity. HRIG: human rabies immunoglobulin; PEP: post-exposure prophylaxis

**Figure 3:**
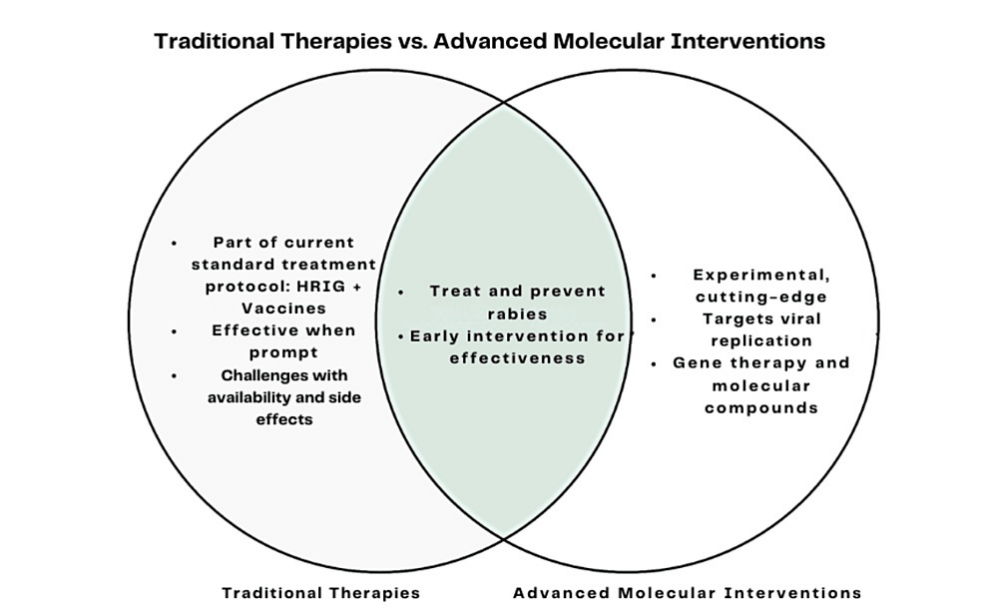
Comparison of the traditional therapies (HRIG + vaccines) with advanced molecular interventions in rabies treatment. Traditional methods are established and prompt, while advanced interventions focus on viral replication and immune response. Both are crucial in early rabies intervention and prevention. HRIG: human rabies immunoglobulin

Furthermore, monoclonal antibodies specific to rabies virus proteins are being investigated for their therapeutic potential, offering a more targeted, precise, and time-sensitive approach to rabies treatment than HRIG [36]. From serum polyclonal antibodies to hybridoma monoclonal antibodies (mAbs) and from murine mAbs to human mAbs, considerable progress has been made in the urgent PEP of rabies [36]. RVC20, a broadly neutralizing mAb, was shown to neutralize all 35 tested RABV strains worldwide [36]. The efficiency of neutralizing mAb cocktails has been confirmed in clinical trials [36]. However, developing a neutralizing rabies cocktail binding to the nonoverlapping glycoprotein epitopes is a challenge, indicating the need for subsequent research. Eventually, safe, effective, and affordable rabies mAbs are presumed to replace HRIG in rabies PEP (Figures 1-3).

Global impact and challenges

Rabies is a neurotropic RNA virus that claims roughly 50,000 lives each year [37]. Although it is spread most often via rabid animals biting humans, and not human-to-human transmission, it is still a major concern as infection almost always leads to death [37]. Because of the enormous toll this virus takes on humankind, governments and nations have taken many measures to ensure disease control and prevention. One of the largest principles for disease prevention is known as One Health.

One Health refers to the collaboration of several disciplines of health, such as veterinarian, environmental, and human, to solve a problem. As of now, the human health discipline has the most to gain from eradicating rabies in canines specifically, since most human deaths due to rabies are caused by a rabid canine bite [37]. However, the human discipline does not use its resources to assist the veterinarian discipline in controlling this disease [37]. A study based in Indonesia researched the effectiveness of a One Health systemic approach to eradicating rabies over the course of 10 years [38]. This study used several strategies to approach disease control, the most significant being mass canine vaccination. They utilized the One Health principle by using government resources to obtain high-quality vaccinations and surveillance for the vulnerable canines on the island. According to the study, a One Health strategy demonstrates promising results when aligned with political will and national commitment [38]. Rabies control programs have achieved success but are hindered by challenges in implementation and accessibility.

Another challenge in managing rabies is the large economic toll it takes on the world. One aspect of this is the cost it takes to administer life-saving PEP. The current regimen consists of up to five clinic visits, with both the vaccination and the transportation costs being high [39]. A study based in southern China tested the efficacy and safety of a 2-1 intramuscular regimen for prophylaxis with the aim of lowering the cost of administration [39]. The results demonstrated no statistically significant difference between the two regimens, meaning that there is potential money to be saved by administering the 2-1 regimen without sacrificing quality [39]. Another barrier for rabies eradication is limited education and public awareness. A study in Azerbaijan tested the effect that public awareness initiatives had on rabies containment [40]. According to the study, those exposed to rabies awareness campaigns are up to four times more likely to vaccinate their pets, a step towards rabies eradication [40]. Additionally, the study portrayed that awareness campaigns improve public knowledge of not only the disease’s symptoms but also vaccination schedules [40]. Based on these studies and the continued loss of life, the economic and societal implications of rabies necessitate cost-effective solutions and comprehensive public awareness campaigns.

## Conclusions

Rabies is a long-standing and lethal viral infection transmitted through animal bites or contact with infected saliva. Louis Pasteur's development of the rabies vaccine in 1885 marked a significant milestone in preventing many cases. The current preventive measures focus on human and animal vaccination, with efforts towards next-generation vaccines. The historical development of rabies vaccines has progressed from inactivated and live-attenuated forms to modern recombinant techniques, each with advantages and limitations. Post-exposure prevention involves a combination of human rabies immunoglobulin (HRIG) and rabies vaccine administration. The standard inactivated rabies virus vaccine remains widely used, with ongoing research exploring novel formulations and adjuvants to enhance efficacy. Gene therapy, virus-like particles, and monoclonal antibodies are emerging as potential advanced treatment options. The pre-exposure prophylaxis vaccination series, administered before potential exposure, is a crucial preventive measure. Exploring novel treatment strategies, including combined vaccine therapies and advanced molecular interventions, provides hope for more effective and accessible treatments, especially for active infections showing symptoms. As the field continues to evolve, a collaboration between researchers, clinicians, and public health authorities is crucial for primarily optimizing the rabies cocktail composition tailored to each PEP patient. Proper initiation of treatment and therapeutic doses should be considered for humans undergoing combination therapy.

Global initiatives, such as the Global Strategic Plan, aim to eradicate human fatalities due to rabid canines by 2030, showcasing the importance of mass vaccination of animals, especially canines. Combining veterinary, environmental, and human health efforts, the One Health approach shows promise in controlling rabies. Challenges include economic barriers to post-exposure prophylaxis, limited education, and public awareness. Cost-effective solutions and comprehensive awareness campaigns are essential for successful rabies eradication strategies. Overall, continued research and collaborative efforts are crucial in the ongoing battle against rabies, a disease that claims almost 100% of the affected individuals' lives if left untreated.
